# Dual action of pyroligneous acid in the eco-friendly synthesis of bactericidal silver nanoparticles

**DOI:** 10.1016/j.heliyon.2022.e11234

**Published:** 2022-10-22

**Authors:** Lúcio C.D. Medeiros, Rafael S. Fernandes, Celso Sant’Anna, Luiz H.S. Gasparotto

**Affiliations:** a Biological Chemistry and Chemometrics Research Group, Institute of Chemistry, Federal University of Rio Grande do Norte, Natal 59072-970, RN, Brazil; b Institute of Chemistry, Federal University of Rio Grande do Norte, Natal 59072-970, RN, Brazil; c Laboratory of Microscopy Applied to Life Sciences - Lamav, National Institute of Metrology, Quality and technology—Inmetro, Duque de Caxias 25250-020, RJ, Brazil

**Keywords:** Environmentally friendly process, Pyroligneous acid, Alkaline synthesis, Silver nanoparticles, Dual action, Antibacterial effect

## Abstract

In the present study, we demonstrate that pyroligneous acid (PA), also known as wood vinegar, functions efficiently as both reducing and stabilizing agent in the synthesis of silver nanoparticles (AgNPs). The synthesis and stabilization of AgNPs take place in the following fashion: 1) in alkaline environment, oxygenated species (phenols in the present case) contained in PA reduce silver ions to metallic silver; 2) acetic acid, abundantly present in PA, adsorb onto the AgNPs conferring electrostatic stabilization. This mechanism is supported by GC-MS and RAMAN analysis, with the former revealing the compounds lacking in PA after nanoparticle synthesis and the latter demonstrating acetic acid adsorbed on the nanoparticles. The AgNPs produced via this method were quite stable up to 150 days (zeta potential = -56 mV). The AgNPs were then found to inhibit the growth of *Escherichia coli* and *Staphylococcus aureus*. Concerning PA, we showed that it displays bactericidal properties only under acidic conditions. This study contributes to the development of more environmentally benign routes to produce nanomaterials.

## Highlights

• Pyroligneous acid as reducing and stabilizing agent to produce silver nanoparticles.
• No hazardous chemicals employed in the synthesis of the nanoparticles.
• Pyroligneous acid displays bactericidal activity only under acidic conditions.
• Mitigation of the environmental impact of pyrolysis gasses.

## Graphical abstract

**Figure:**
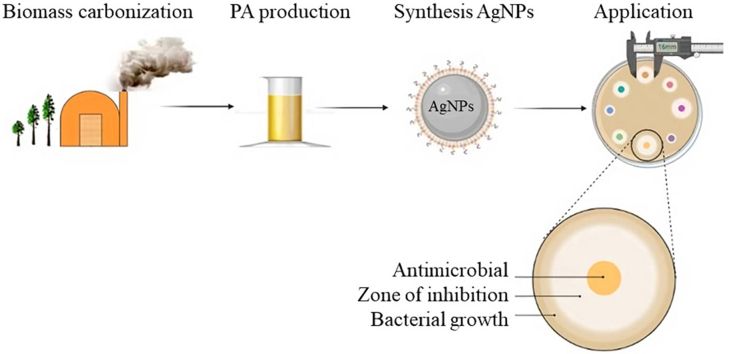

## 1 Introduction

Promotion and adoption of renewable natural resources have been worldwide efforts to mitigate global environmental and health issues [1, 2]. An interesting approach to tackle this problem is the conversion of plant biomass into gaseous, liquid, and solid fuels by various processes such as carbonization, gasification, and pyrolysis [3, 4]. The latter, amongst charcoal and smoke, produces a highly oxygenated organic liquid known as pyroligneous acid (PA).

PA, also called wood vinegar, consists mainly of water and acetic acid and contains a myriad of organic chemical compounds (guaiacols, catechols, syringols, phenols, vanillins, furans, pyrans, carboxaldehydes, hydroxyketones, sugars, alkyl aryl ethers). In agriculture, not only has PA been found to enhance plant growth [5, 6] and fruit size [7], it has also been shown to inhibit the proliferation of plant pathogens such as fungi and bacteria [8, 9]. In the realm of nanotechnology, an application hypothesized for PA is the reduction-agent role in the synthesis of noble metallic nanoparticles. As demonstrated by Gomes et al. [10], a compound must have a hydroxyl group prone to deprotonation to reduce gold and silver ions into their respective nanoparticles in alkaline media. Eligible functional groups are alcohols, aldehydes, and ketones (the latter generates a hydroxyl under alkaline conditions). The formed alkoxide (deprotonated hydroxyl) is then the moiety responsible for the reduction of the noble ions into their zero-valent counterparts. Given that PA has a plethora of hydroxyl-containing molecules [11] (aldehydes, carbohydrates, alcohols, and phenols), it is conceivable that PA could function as a reducing agent in alkaline conditions.

In the present study, PA was employed for the first time as a reducing agent in the alkaline synthesis of silver nanoparticles (AgNPs). An important advantage of using PA is that no other agent was required to stabilize the AgNPs in solution. As discussed later, acetate ions, which are quite abundant in neutralized PA, adsorb onto AgNPs conferring them a negative charge that contributes to the overall stabilization. We also demonstrated via gas chromatography-mass spectrometry (GC-MS) that many compounds present in PA disappear from the solution after the synthesis of AgNPs, suggesting that they functioned as reducing agents in the process. Finally, the AgNPs were found to inhibit the growth of *Escherichia coli* and *Staphylococcus aureus*. It was also discovered that PA displays bactericidal properties only under acidic conditions, a result also discussed throughout the text. This study contributes to the development of more environmentally benign routes to produce nanomaterials.

## 2 Materials and methods

### 2.1 Synthesis and characterization of PA

The synthesis, purification, and characterization of PA from *Eucalyptus urograndis* are fully described elsewhere [12]. Briefly, wood wedges were dried and then carbonized in a muffle furnace (SP LABOR/1200DM/G), with pyrolysis gases trapped and condensed to a brown liquid. Afterwards, the liquid fraction was bidistilled at 20 mmHg and 100 °C to yield a pale-yellow liquid that was then analyzed via CG-MS.

### 2.2 Synthesis of AgNPs

A typical synthesis proceeds as follows: a mixture of 171.0 mL of water and 9.0 mL of AgNO_3_ (20.0 mmol L^−1^) is produced in a beaker (namely beaker 1). In a separate beaker (beaker 2), 158.4 mL of water are mixed with 10.8 mL of AP and 10.8 mL of NaOH (1.0 mol L^−1^). The content of beaker 2 was poured into beaker 1 to produce a dark-brown solution, which is indicative of AgNPs formation. The resulting solution was then centrifuged at 10.000 rpm for 5 min and redispersed in 360 mL of water. A 2^3^ full factorial design of experiments was implemented to check the influence of the parameters shown in Table 1, with the full width at half maximum (FWHM) of UV-vis spectra employed as the response. Additionally, AgNPs stabilized with acetate ion were also produced for comparison. To that end, beakers 1 and 2 contained 4.550 mL of water +0.150 mL of AgNO_3_ (20 mmol L^−1^) + 0.300 mL of sodium acetate (0.26 mol L^−1^) and 1.0 mL of glycerol (1.00 mol L^−1^) + 0.300 mL of NaOH (1.00 mol L^−1^), respectively. The content of beaker 2 was added to beaker 1 to produce AgNPs stabilized by acetate.

**Table 1:** Full factorial experimental design layout.

Factor	(-)	(+)
A–Agitation (min)	0∗	1∗
B–Temperature (º C)	25	60
C–[Ag] (mmol L^−1^)	0.30	0.50

∗ 0 and 1 mean with and without agitation, respectively.

### 2.3 Characterization of AgNPs

UV-vis absorption spectra were acquired with a Shimadzu 1800 UV-Vis Spectrophotometer, Japan. Dynamic light scattering (DLS) and zeta potential analyses were conducted in a NANO-flex 180° DLS (Colloid Metrix). Transmission electron microscopy (TEM) was carried out in a FEI Tecnai G2 Spirit BioTWIN microscope operating at 120 kV. Raman measurements were acquired in a Raman Horiba spectrometer (LabRAM HR Evolution) equipped with the Olympus BX41 open microscope stage (objective 50×). Spectra were recorded at an excitation wavelength and power of 785 nm and 45 mW, respectively. Each spectrum was a product of ten scans accumulated at an integration time of 5 s in the 200–2000 cm^−1^ range with a grating of 600 lines/mm.

### 2.4 GC-MS analyses

In order to discover the compounds in PA responsible for reducing silver ions to zero-valent silver, AgNPs solutions were centrifuged, and the supernatant subjected to CG-MS. Initially, a PA solution containing 282.0 mL of water +9.0 mL of AP + 9.0 mL of NaOH (1.0 mol L^−1^) was set as the standard to be compared with the supernatant of AgNPs. 300 mL were taken from both the PA solution and supernatant and had their pH adjusted to 5. Afterwards, 5 mL of dichloromethane were added to both aliquots to extract the organic compounds, with 2 mL of the organic phase reserved for GC-MS analysis.

CG-MS experiments were carried out in a SHIMADZU GC-2010-PLUS spectrometer equipped with a SH-Rtx-5MS column (30 m × 0.25 mm) under the following parameters: ultrapure helium (99.9995%) as carrier gas at 1.0 mL min^−1^; sample volume of 1 μL at a split ratio of 1:100; inlet temperature = 280 °C; ion source temperature = 230 °C; column temperature = 40 °C; heating rate of 5 °C min^−1^ until 180 °C, where kept for 2.0 min; heating rate of 7 °C min^−1^ until 260 °C, where kept for 5 min.

### 2.5 Bacteriological experiments

*Escherichia coli* (ATCC 25922) and *Staphylococcus aureus* (ATCC 25923) were cultured in Müller-Hinton agar medium on Petri dishes at 37 °C for 24 h. Then bacteria colonies were transferred into a 0.9 % saline solution to yield a suspension with a turbidity of 0.5 MaCFarland (1 × 10^8^ UFC mL^−1^). The suspension was then swabbed onto another Petri dish containing the Müller-Hinton agar medium. Afterwards, 6-mm wells were made in the culture medium and filled with 50 μl of the antimicrobial agents (AgNPs and AP). Gentamicin and pure water were employed as negative and positive controls, respectively. The plates were incubated at 37 °C for 24 h and had their inhibition zones expressed in the millimeter unit.

## 3 Results and discussion

### 3.1 Synthesis of AgNPs with PA

**Figure 1:**
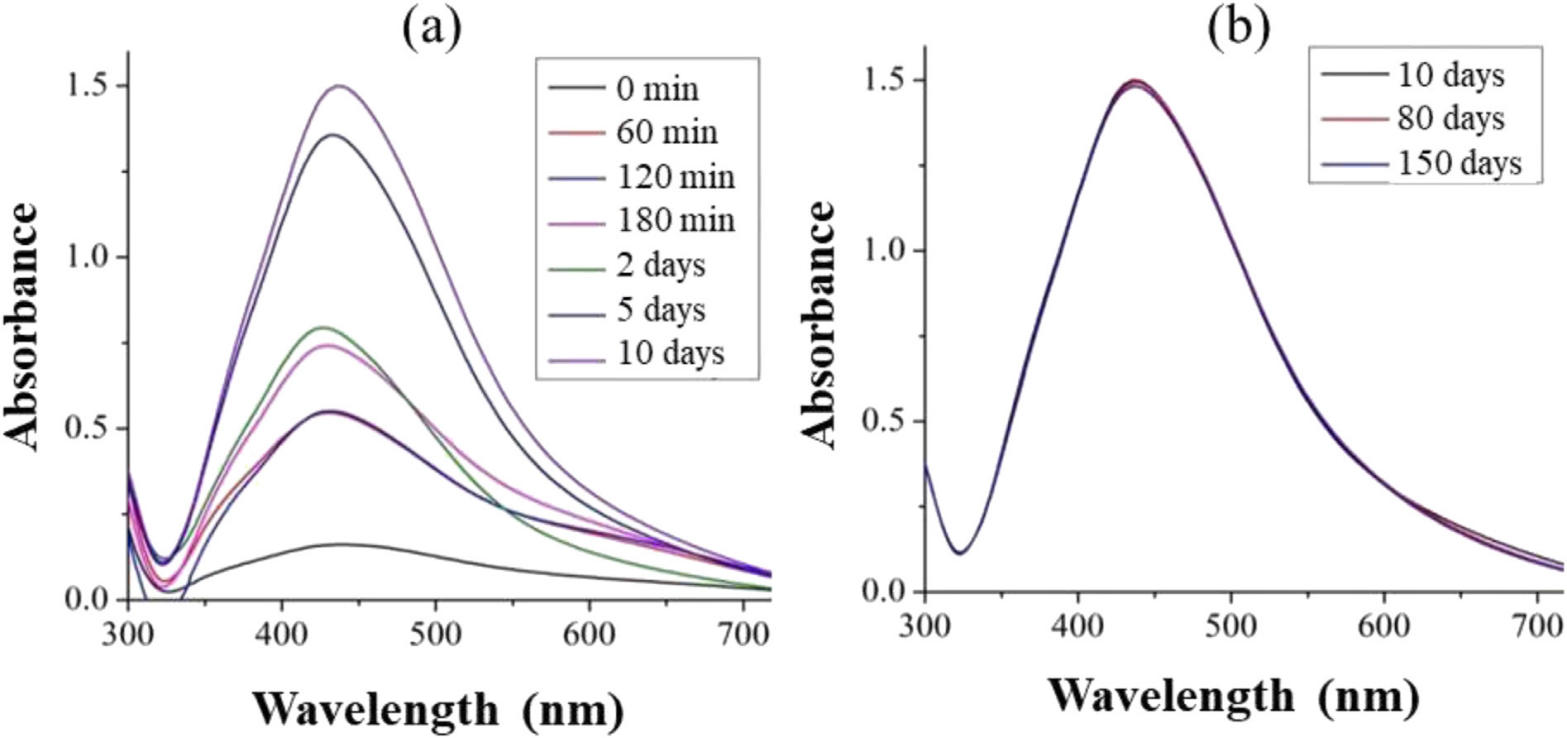
(a) Time-evolution of UV-vis spectra acquired for AgNPs synthesized with PA as reducing and stabilizing agents; (b) stability data registered up to 150 days. Condition of synthesis: [Ag] = 0.5 mmol L^−1^, [PA] = 3 % (v/v) and [NaOH] = 30 mmol L^−1^.

**Figure 2:**
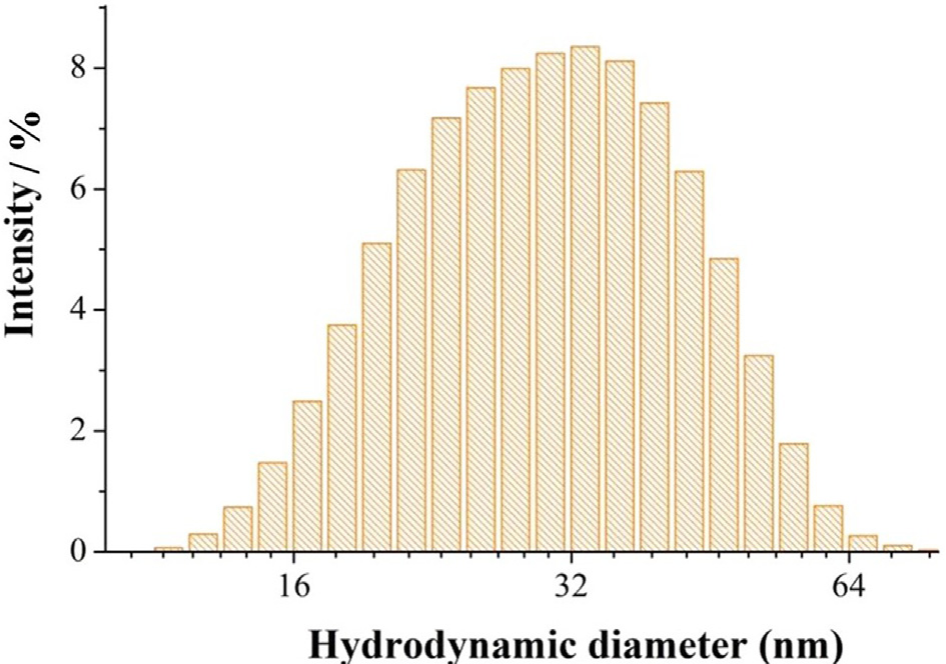
DLS fo for AgNPs synthesized with PA as reducing and stabilizing agents. Condition of synthesis: [Ag] = 0.50 mmol L^−1^, [PA] = 3 % (v/v) and [NaOH] = 30 mmol L^−1^.

**Figure 3:**
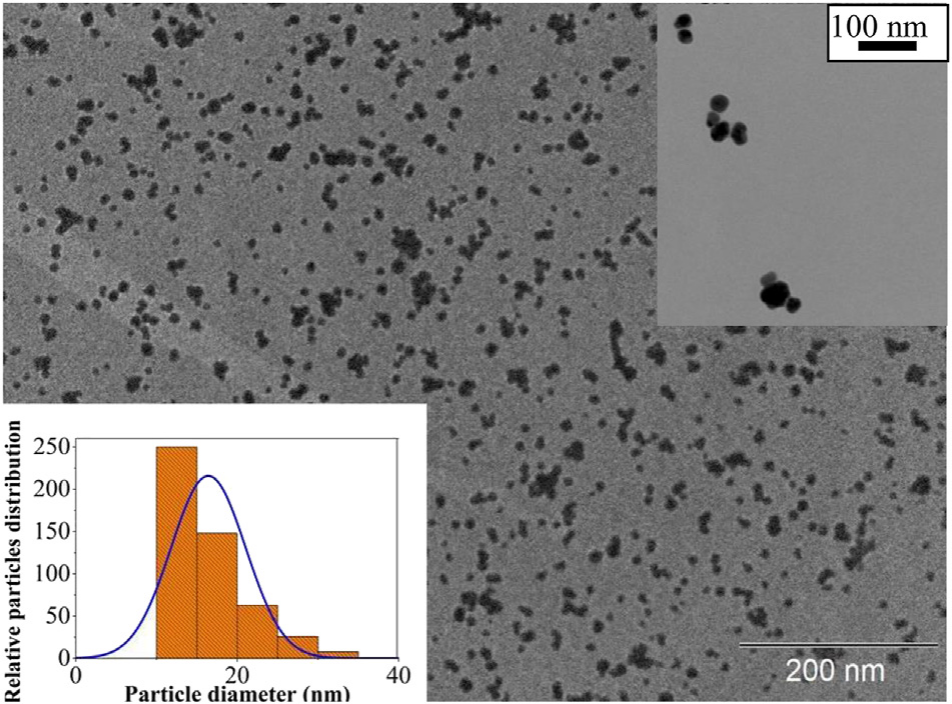
TEM image of AgNPs synthesized with PA. Condition of synthesis: [Ag] = 0.5 mmol L^−1^, [PA] = 3 % (v/v) and [NaOH] = 30 mmol L^−1^. *Inset*: higher-magnification TEM image of the AgNPs.

The synthesis of PA yielded a pale-yellow smoky-odor liquid with density and pH of 1.05 g cm^−3^ and 2.80, respectively. These characteristics agree with those found by Campos [13], Pimenta et al. [14], and Grewal et al. [15]. Afterwards, AgNPs were synthesized via reduction of Ag^+^ by PA in alkaline medium. UV-vis spectroscopy revealed the evolution of a band that peaked at 420 nm upon mixing alkaline PA with AgNO_3_, an indicative of AgNPs formation. The yellow color emerges from the surface plasmon band (SPB) resulting from the resonant coherent dipolar oscillations of the electron gas (electrons of the conduction band) at the surface of nanoparticles [16]. The intensity of the band increased during the following 10 days (Figure 1a), remaining stable up to 150 days (Figure 1b). The zeta potential of the solution was -56.0 mV, attesting its excellent stability. The hydrodynamic diameter observed via dynamic light scattering (DLS) was 33.5 nm (Figure 2), while TEM probed from diameters from 16 nm to 30 nm depending on the silver ion concentration (Figure 3). XRD of the product (not shown) confirmed the material to be elemental silver. The discrepancy in sizes probed by DLS and TEM is due to the fact that DLS measures the hydrodynamic size of particles, meaning that the adsorbed stabilized agent is also computed for the overall size [17]. The AgNPs synthesis was optimized via an experimental design with stirring (A), temperature (B), and silver ion concentration (C) as factors and the full width at half maximum (FWHM) as response. FWHM is the measurement of bandwidth of a Gaussian like-curve which correlates with particle size distribution [18]. Small FWHM values indicate narrower size distribution. Experiments were performed in triplicate with mean values for FWHM (X_m_) displayed in Table 2.

**Table 2:** Coded values and FWHM responses for the full factorial experimental design layout.

Treatment	A	B	C	A.B	A.C	B.C	A.B.C	X_m_
1	−	−	−	+	+	+	−	92.97
2	+	−	−	−	−	+	+	111,08
3	−	+	−	−	+	−	+	103.71
4	+	+	−	+	−	−	−	114.21
5	−	−	+	+	−	−	+	100.43
6	+	−	+	−	+	−	−	118.87
7	−	+	+	−	−	+	−	100.70
8	+	+	+	+	+	+	+	145.61

A–Stirring, B–Temperature, C–silver ion concentration, X_m_–mean values for FWHM.

**Figure 4:**
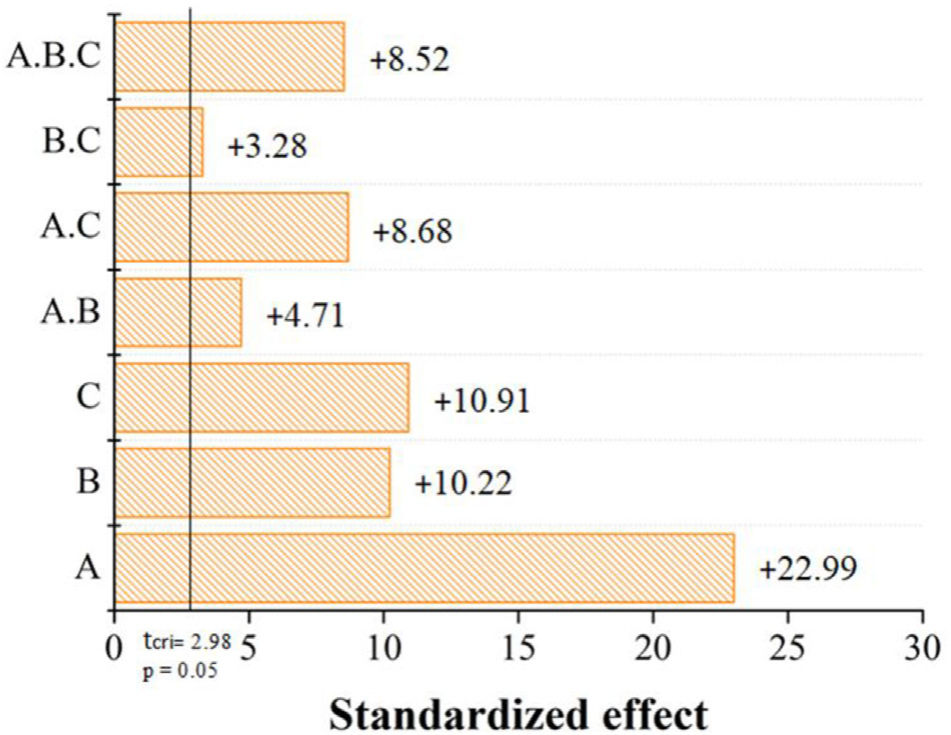
Pareto chart showing influence of the studied variables: A–Stirring, B–Temperature, C–silver ion concentration.

As seen in Figure 4, all factors and their interactions were statistically significant to the size distribution of AgNPs. Furthermore, upon their increase (from (−) to (+)) all factors led to augmentation of FWHM. Based on Table 2 and Figure 4, treatment 1 provided the lower value for FWHM, which translates into a narrower size distribution. The Pareto chart (Figure 4) revealed the variable “stirring” to impact the FWHM the most, showing that smaller particles were produced under no-stirring conditions. This effect may be explained by the lower rate of reagent collision and mass transfer that limit the growth of silver nuclei [19]. Broader plasmon bands were observed upon increasing both the temperature and silver ion concentration, which could be explained by an altered reaction kinetic and aggregative mechanisms of the nanoparticles according to the classical LaMer mechanism [20, 21].

### 3.2 Mechanism of Ag^+^ reduction and nanoparticle stabilization by PA in alkaline medium

**Figure 5:**
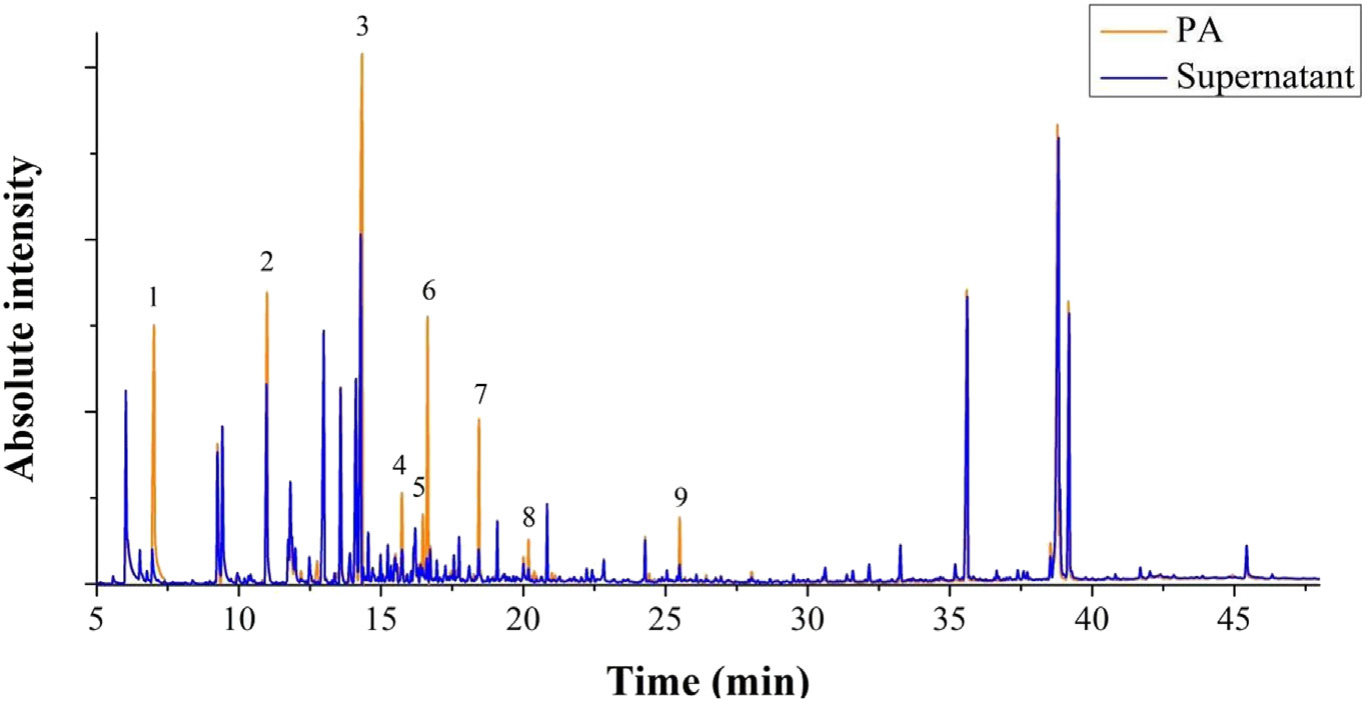
GC-MS of PA solution and the supernatant from the synthesis of AgNPs produced with PA.

In a study by Gomes et al. [10], it was demonstrated that alkoxides formed from any hydroxyl-containing molecules function as reducing agents in the formation of silver and gold nanoparticles. At high pH alkoxides can be formed from alcohols, aldehydes, and ketones, explaining therefore the capability of a myriad of chemicals to reduce gold and silver ions. In the case of PA, it is well documented it contains a plethora of oxygenated compounds such as guaiacols, catechols, syringols, phenols, carboxaldehydes, hydroxyketones, and sugars. In order to identify the chemical compounds involved in the formation of silver nanoparticles, GC-MS was conducted on the PA solution before and after the synthesis of AgNPs, with results shown in Figure 5. As seen, in both chromatograms most peaks displayed similar intensities, suggesting that those compounds were not involved in the formation of AgNPs. On the other hand, nine peaks had their intensities severely decreased after the synthesis of AgNPs. The compounds relative to the suppressed peaks were mainly phenols (Table 3), which are known to produce phenolate ions in basic media and then reduce silver ions [22], in accordance with the results obtained by Gomes et al. [10]. It is thus conceivable that those compounds participated in the reduction of silver ions.

**Table 3:** Main compounds identified in PA.

Peak	Retention time (min)	Compound	Molecular formula	Average mass	Similarity (%)
1	7.020	Furfural	C_5_H_4_O_2_	96.084	98
2	10.995	5-Methylfurfural	C_6_H_6_O_2_	110.111	98
3	14.334	Guaiacol	C_7_H_8_O_2_	124.137	97
4	15.728	2.4-Xylenol	C_8_H_10_O	122.164	86
5	16.470	o-Creosol	C_7_H_8_O	108.138	96
6	16.638	p-Creosol	C_7_H_8_O	108.138	96
7	18.444	4-Ethylguaiacol	C_9_H_12_O_2_	152.190	97
8	20.185	4-Propylguaiacol	C_10_H_14_O_2_	166.217	90
9	25.498	Furan-2-carbaldehyde, (N0-nitroamidino) hydrazone	C_6_H_7_N_5_O_3_	197.054	82

**Figure 6:**
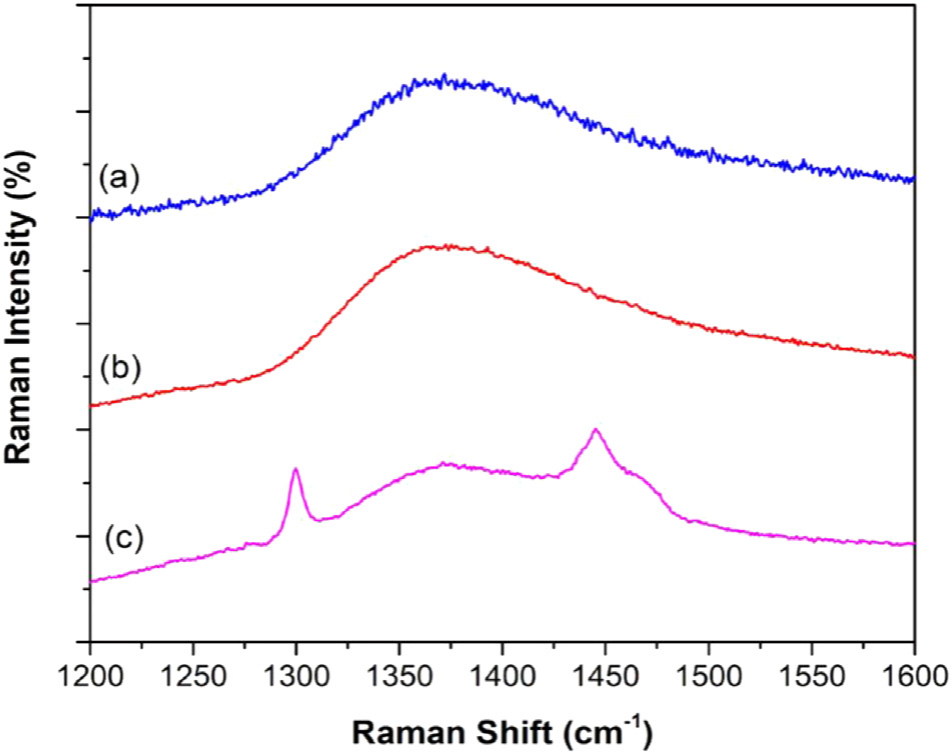
Raman spectra for (a) acetate solution, (b) AgNPs stabilized by acetate and (c) AgNPs + PA.

**Figure 7:**
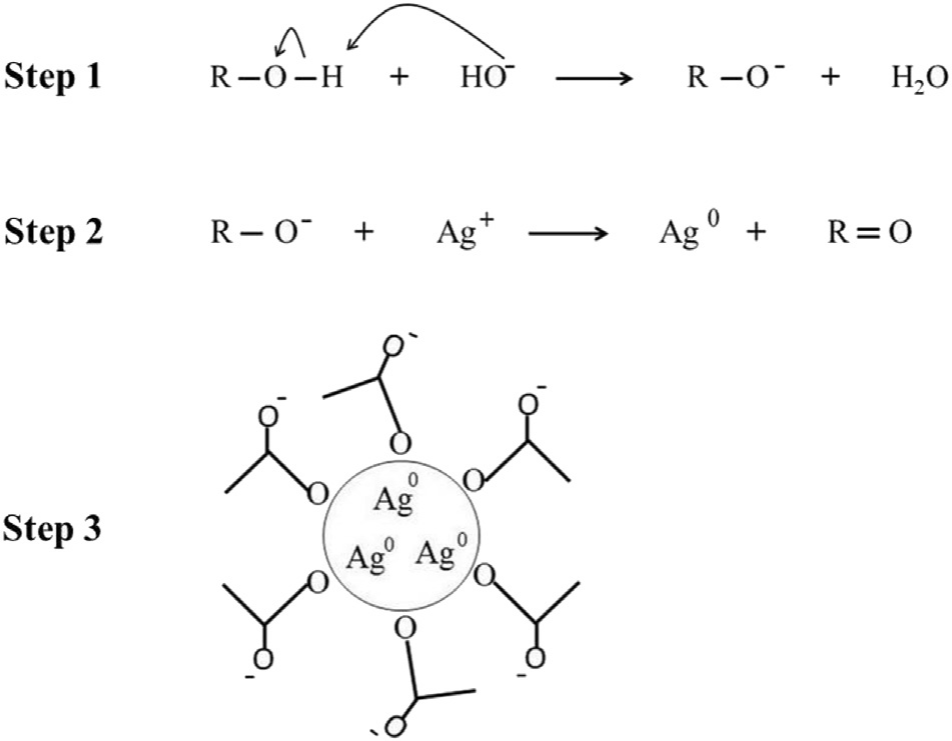
Schematic representation of the AgNPs synthesis and stabilization by PA.

Concerning the stabilization of AgNPs, Raman spectroscopy revealed that the acetate ion plays a major role in preventing aggregation. Figure 6 shows Raman spectra acquired for (a) acetate solution, (b) AgNPs stabilized by acetate, and (c) AgNPs produced by PA and redispersed in ultrapure water after centrifugation. All spectra displayed a broad band centered at 1380 cm^−1^ assigned to the symmetrical elongation of C–O of the acetate [23], suggesting that acetate plays a pivotal role in the stabilization of AgNPs produced by PA. This result is corroborated by the zeta potential of -56 mV, meaning that negatively charged molecules (acetate in the present case) contribute to the overall stabilization via electrostatic repulsion. Additionally, two other bands at 1300 cm^−1^ and 1450 cm^−1^ appeared for centrifuged AgNPs. These bands are related to the symmetric and anti-symmetric C=O stretching vibration of carboxylic groups [24] in furan-2-carbaldehyde (Table 3). This result suggests that, in addition to acetate, furan-2-carbaldehyde is also adsorbed onto AgNPs contributing to the overall stabilization. Figure 7 presents a scheme of the AgNPs formation.

### 3.3 Antimicrobial susceptibility tests

**Figure 8:**
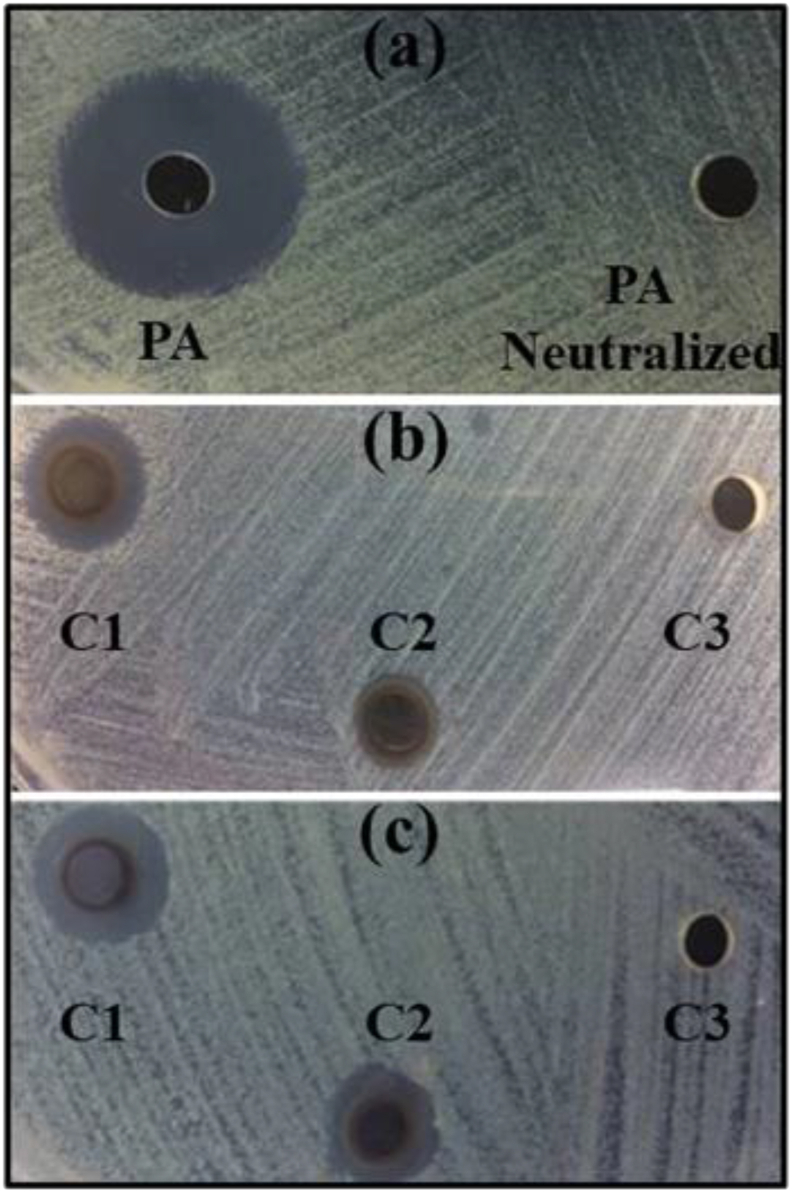
Photographs of inhibition zones for (a) *E. coli* + PA, (b) *E. coli* + AgNPs, and (c) *S. aureus* + AgNPs. Silver loading: C1 = 38 μg; C2 = 18 μg; C3 = 1.9 μg.

Firstly, we investigated the bactericidal potential of PA. Although it has been reported to display bactericidal properties [25, 26], they strongly depend on pH and whether PA was subjected to further purification [27]. In line with results of Medeiros and Gasparotto [28], herein PA was active against both strains of bacteria only at low pH, with the inhibitory activity vanishing completely upon neutralization (Figure 8A). It becomes clear that the variety of compounds in PA, at their typical concentrations, are not responsible for any bactericidal activity. As acetic acid is substantially more concentrated in PA than other compounds (guaiacol, phenols, and furfural), under non-neutralized conditions acetic acid is then responsible for the observed inhibitory activity. Inhibition halos for *E. coli* and *S. aureus* after exposition to different AgNPs loadings are presented in Figure 8B and 8C and Table 4. It is important to stress that the AgNPs solution was neutralized to access the sole AgNPs inhibitory potential. As observed, both bacteria strains were susceptible to the AgNPs. Moreover, as expected, larger inhibition halos were observed with increasing silver load. When compared with previous studies, the AgNPs reported herein produced halos of the same size or even larger at similar silver loadings, as seen in Table 5.

**Table 4:** Values of inhibition zones for (a) *E. coli* + AgNPs, (b) *S. aureus* + AgNPs, (c) *E. coli* + PA. Silver concentrations: C1 = 38 μg; C2 = 18 μg; C3 = 1.9 μg.

Inhibition zone diameter (mm)		
	*E. coli*	*S. aureus*
PA	17.7 (±0.6)	17.0 (±1.0)
PA neutralized	0	0
C1	12.7 (±1.2)	12.0 (±1.0)
C2	9.7 (±0.6)	10.3 (±1.2)
C3	0	0
Control (+)	22.7 (±0.6)	22.3 (±0.6)
Control (−)	0	0

**Table 5:** Inhibition activities (in terms of halo size) reported in the literature and in the present work.

	Silver loading (μg)	Halo (mm)	Reference
*E. coli*	125	15.0	[30]
	100	14.0	[31]
	54	11.6	[29]
	20	10.9	[32]
	14	9.0	[35]
	10	8.0	[34]
	38	12.7	This work
*S. aureus*	125	17.0	[30]
	100	14.0	[31]
	54	9.2	[29]
	25	12.0	[33]
	14	11.0	[35]
	10	8.0	[34]
	38	12.0	This work

It has been demonstrated that at the physiological pH (around 7) PA displays no inhibitory activity towards *S. Aureus* and *E. coli*. Hence, at that condition the observed activity is entirely dictated by the AgNPs. Despite the exact mechanism of silver nanoparticles’ antibacterial activity is yet to be fully clarified, some suggestions have been put forward [36]. AgNPs can kill microbes by continually releasing silver ions [37] that adhere to the cell wall and cytoplasmic membrane as a consequence of electrostatic attraction and affinity to sulfur proteins. The adhered ions can, in turn, render the cytoplasmic membrane more permeable, facilitating the disruption of the bacterial envelope [38]. Reactive oxygen species are regarded as the main agent in the cell membrane disruption and deoxyribonucleic acid (DNA) alteration. Moreover, silver ions are inhibitors of the synthesis of proteins by denaturing ribosomes in the cytoplasm [39].

It has also been demonstrated that zero-valent silver can kill bacteria [40], as the accumulated AgNPs culminate in cell membrane denaturation. AgNPs are also capable of penetrating bacterial cell walls and subsequently altering the morphology of the cell membrane. The result is the denaturation of cytoplasmic membrane, rupture of organelles, and ultimately cell lysis. Additionally, AgNPs can lead to cell apoptosis and halt cell multiplication by disturbing the phosphorylation of protein substrates [41].

## 4 Conclusion

Silver nanoparticles were successfully synthesized with pyroligneous acid in alkaline media. GC-MS analyses suggest phenols to be responsible for the reduction of silver ions to zero-valent silver. Concerning the stabilization of AgNPs, Raman spectroscopy allied with zeta potential revealed acetate ions to adsorb onto the surface of AgNPs, hence ensuring electrostatic repulsion. AgNPs produced via the PA method were found to be quite stable up to 150 days. It has been demonstrated that PA inhibits the growth of *Escherichia coli* and *Staphylococcus aureus* only at low pH (∼3.0), evidencing that acetic acid is accountable for the stand-alone PA activity. At the physiological pH the AgNPs produced herein displayed bacteriostatic activity similar to those reported in the literature. Using PA in the synthesis of nanomaterials is an interesting approach to avoid the release of volatile chemicals and tars into the atmosphere.

## Declarations

### Author contribution statement

Lúcio C. D. Medeiros: Conceived and designed the experiments; Performed the experiments; Analyzed and interpreted the data.

Rafael S. Fernandes: Performed the experiments; Analyzed and interpreted the data.

Celso Sant’Anna: Performed the experiments; Analyzed and interpreted the data; Contributed reagents, materials, analysis tools or data.

Luiz H. S. Gasparotto: Conceived and designed the experiments; Analyzed and interpreted the data; Contributed reagents, materials, analysis tools or data; Wrote the paper.

### Funding statement

This work was supported by Coordenação de Aperfeiçoamento de Pessoal de Nível Superior.

### Data availability statement

Data included in article/supplementary material/referenced in article.

### Declaration of interests statement

The authors declare no conflict of interest.

### Additional information

No additional information is available for this paper.
